# Ethical considerations in forensic genetics research on tissue samples collected post-mortem in Cape Town, South Africa

**DOI:** 10.1186/s12910-017-0225-6

**Published:** 2017-11-29

**Authors:** Laura J. Heathfield, Sairita Maistry, Lorna J. Martin, Raj Ramesar, Jantina de Vries

**Affiliations:** 1 Division of Forensic Medicine and Toxicology, Department of Pathology, Faculty of Health Sciences, University of Cape Town, Anzio Road, Observatory, Cape Town, 7925 South Africa; 2 MRC/UCT Human Genetics Research Unit, Division of Human Genetics, Department of Pathology, Institute of Infectious Diseases and Molecular Medicine, Faculty of Health Science, University of Cape Town, Anzio Road, Observatory, Cape Town, South Africa; 3 Department of Medicine, Faculty of Health Sciences, University of Cape Town, Anzio Road, Observatory, Cape Town, 7925 South Africa

**Keywords:** Vulnerable group, Next of kin, Sudden unexpected death of infants, Suicide, Forensic, Post-mortem, Medico-legal, Biomedical, Research ethics, South Africa

## Abstract

**Background:**

The use of tissue collected at a forensic post-mortem for forensic genetics research purposes remains of ethical concern as the process involves obtaining informed consent from grieving family members. Two forensic genetics research studies using tissue collected from a forensic post-mortem were recently initiated at our institution and were the first of their kind to be conducted in Cape Town, South Africa.

**Main body:**

This article discusses some of the ethical challenges that were encountered in these research projects. Among these challenges was the adaptation of research workflows to fit in with an exceptionally busy service delivery that is operating with limited resources. Whilst seeking guidance from the literature regarding research on deceased populations, it was noted that next of kin of decedents are not formally recognised as a vulnerable group in the existing ethical and legal frameworks in South Africa. The authors recommend that research in the forensic mortuary setting is approached using guidance for vulnerable groups, and the benefit to risk standard needs to be strongly justified. Lastly, when planning forensic genetics research, consideration must be given to the potential of uncovering incidental findings, funding to validate these findings and the feedback of results to family members; the latter of which is recommended to occur through a genetic counsellor.

**Conclusion:**

It is hoped that these experiences will contribute towards a formal framework for conducting forensic genetic research in medico-legal mortuaries in South Africa.

## Background

The field of forensic genetics encompasses the analysis of deoxyribonucleic acid (DNA) from human remains for the purposes of identifying an individual or to help understand their cause of death [1, 2]. Although the discipline has a strong service-orientated focus, research and development play an important role in the improvement of this evidence-based practice. Such research requires the use of genetic material obtained from body fluids and tissue, but the use of post-mortem material in research is a sensitive subject. This is because taking informed consent from grieving family members needs to be strongly justified; there is the potential to do more harm than good and to exploit a vulnerable population. In this manuscript, we will describe some of the most pertinent ethical challenges that we encountered in our ongoing forensic genetics research.

The Division of Forensic Medicine and Toxicology (DFMT) at the University of Cape Town (UCT) leads an active role in service delivery, teaching and research activities. UCT’s DFMT is closely affiliated with the Department of Health, Western Cape Government, which oversees the post-mortem investigation of individuals whose deaths are deemed unnatural in the Western part of the Cape Metropole of South Africa. Autopsies have been performed for centuries and serve to establish the nature and extent of underlying natural disease, the process and mechanism leading to death, and to enable learning, teaching and research [3]. Post-mortem investigations, from which we derive our genetic research samples, are conducted at the Salt River Mortuary, which conducts more than 3500 post-mortem examinations each year. The DFMT hosts various research projects together with those of the present authors.

### Summary of studies

Over the past three years, we initiated two projects that involve the use of post-mortem specimens for prospective genetic analyses. The first involves an investigation of the epidemiology, genetics and neurobiology of suicide. This study seeks to identify multifactorial contributions to suicidal behaviour. Part of this project involved the pilot prospective collection and genetic testing of biological material from suicide victims to establish whether single nucleotide polymorphisms in candidate genes could predispose a person to commit suicide. This study completed pilot sample collection in October 2015 and obtained informed consent to collect brain tissue and/or blood from 37 out of 40 (92.5%) families during a one-year period.

The second study aims to explore the molecular basis of sudden unexpected death in infants (SUDI) at Salt River Mortuary. SUDI cases undergo post-mortem investigation in an attempt to establish cause of death. Most of these cases at Salt River Mortuary end up being classified as natural, with the leading cause of death being infection [4]. However, some deaths remain undetermined and are thus classified as sudden infant death syndrome (SIDS); which is defined when infants less than one year old die suddenly and unexpectedly, and the cause of death cannot be determined after thorough post-mortem investigation, including a full autopsy, death scene investigation and clinical history review [5].

Common risk factors for SUDI are typically environmental, but despite awareness campaigns about these risk factors in developed countries, there remains a residual burden of SUDI which suggests a genetic basis for at least a component of these deaths [6]. This has led to genetic testing in the form of molecular autopsies to try and classify the cause of death, particularly in SIDS cases [7]. These have taken on the form of genotyping known mutations, sequencing candidate genes or massively parallel sequencing of exomes and genomes. Internationally, this has shown potential in not only classifying the direct genetic cause of death, but also establishing genetic factors that could predispose the infant during their critical stage of development [7–11]. This project focuses on exploring whether specific genetic variants identified elsewhere are also found in our population. The first sample collection phase for the SUDI project was between March 2016 and June 2016, where 38 families out of 40 (95%) gave informed consent for blood and/or buccal swabs to be obtained from their deceased infants for molecular analysis. Thus together, the researchers have spoken to a total of 80 families during the pilot collection phases.

These two studies are among the first prospective molecular studies on deceased individuals conducted in South Africa and as such are novel both to the research regulatory infrastructure (including ethics committees) and to the forensic services. One important secondary function of these research projects is their role in establishing the feasibility and acceptability of carrying out genetic research using post-mortem samples in South Africa. While guidelines exist with regard to research of this nature, most have limited applicability to low- and middle- income countries (LMICs), where the access to resources is severely limited [12, 13]. When speaking to the 80 families of deceased individuals about collecting samples for genetics research, and in seeking regulatory approval for these studies, we noted some remarkable similarities in our experiences, which are described and discussed in the current manuscript.

### Ethical considerations

As we planned and conducted our research, we encountered a number of ethical challenges, some of which were anticipated and others that were not. To our surprise, when looking to literature for guidance about how to address these challenges, we found a scarcity of resources to help us make sense of these challenges.

### Institutional review board: Seeking approval

One of the first and arguably most challenging issues we faced related to seeking ethics approval from our Human Research Ethics Committee (HREC) for these studies. In particular, when the first study was proposed – which was also one of the first ever genetic studies on autopsy samples reviewed by our Institutional Review Board – we faced a barrage of critical questions that took over two months to resolve. These included questions surrounding feedback of results, incidental findings and the timing of the consent, all of which are discussed in this article. At the best of times, genomic research raises fears and anxieties for ethics committees in Africa, relating importantly to the potential for exploitation and concerns about identifiability [14]. We suspect that the novelty of this research, combined with taboos surrounding both suicide and SUDI and the fear that grief could influence individuals’ ability to give informed consent, made our HREC particularly anxious about the proposed projects. We resolved this challenge by establishing a relationship with the ethics committee which allowed for less formal conversations and explanations to occur, specifically regarding the legal and judicial mandates under which medico-legal autopsies are performed in the FPS in South Africa. Through this relationship, we worked with selected members of the HREC to design a workable consent strategy for this study. We also indicated that we would continue to engage with the HREC about our experiences and alert them to any particular challenges that may occur.

### Informed consent: Process of procurement

Since both of these projects required samples to be collect at the autopsy, informed consent needed to be obtained prior to the autopsy taking place. According to the South African National Health Act (Act 61 of 2003) and the Inquests Act (Act 58 of 1959), it is mandated that all deaths deemed to be unnatural are referred for a medico-legal autopsy, and during this investigation, samples may be retained at the discretion of the forensic pathologist, for the purpose of determining cause of death.

Suicide, which is the intentional taking of one’s own life [15], is considered an unnatural death, and therefore requires a mandatory autopsy. SUDI, on the other hand, is referred to for medico-legal autopsy under the circumstances that the death was sudden and unexpected (National Health Act (Act 61 of 2003)); however, the protocols for the post-mortem investigation of SUDI are not standardised in South Africa [16, 17]. In the case of autopsies, the South African laws allow for the obtaining of tissue samples to determine cause of death, but not for research purposes. Hence for these studies to occur, informed consent must be obtained by the individual concerned during their lifetime (or by a minor’s legal guardian), or by their next of kin if the individual is already deceased, to retain and use tissue for research purposes [3]. This raises genuine ethical concerns which need to be acknowledged and addressed.

There were also a number of important differences in the consent processes for the two genetic studies we conducted. The first of these is that while parents of infants typically have legal authority to make decisions for their infants, next-of-kin of adults do not normally have such authority; although de facto they do make treatment and/or research enrolment decisions on behalf of their family members in some selected instances (e.g. in the case of unconsciousness). In that case, next of kin are approached on the assumption that they are best-placed to make decisions on behalf of the individual and may even have been made aware of the individual’s decisions. In other words, for infants, we are asking parents to make a decision based on their own belief system whilst for next of kin of adults we are asking them to make a decision based on what they think the deceased suicide victim would have wanted.

The second important difference relates to the ease with which samples would be obtained during the autopsy. In the case of suicide, autopsies are legally mandated (because the cause of death is considered ‘unnatural’) and samples may be obtained for ancillary testing in any case. Seeking an additional sample for research in that process is fairly straightforward. Not so in the case of SUDI. Without evidence of ‘unnatural’ causes of death, the pathologist is not legally mandated to establish the exact cause of natural death and thus may not need to perform an internal/full autopsy. In that case, the obtaining of a sample may constitute the only surface violation of the corpse, and in the case of very young infants, may require opening up the body to obtain a blood sample from the heart. Since this sample would not have been taken for cause of death determination, there is no legal mandate to take such a sample without consent.

We based the design of our consent strategy on three sources: discussions with the forensic pathology team and the pathologist conducting the suicide genetic study; discussions with the HREC; and on work reported by Odendaal and colleagues [13]. Following these discussions, we agreed that the most appropriate time to obtain informed consent would be before the identification process in a private designated interview. Family members would normally only come to the mortuary once, and for reasons of feasibility and cost, it was important that we approached them at that point. The identification process entails the formal identification of the deceased by the next of kin for legal purposes, and is typically an emotional process, accompanied by paperwork. The researcher/s introduced themselves to the next of kin of the deceased before the identification process and expressed condolences to the family. Next the researcher explained the project, which was also collated into a written ‘information form’ which described the purpose, background and procedure of the research study. This form was approved by the Institutional Review Board and contained the contact details of its chair and of the researchers. Family members could participate voluntarily, and if they decided not to, there was no consequence whatsoever – this was clearly explained to the next of kin. The most common question asked by the next of kin was if participating would delay the autopsy process and eventual collection of their relative for the funeral. It was explained to the next of kin that the collection of additional samples would only take a few extra minutes. Parents of infants who had demised suddenly and unexpectedly were given the option to only agree to a sample being taken if the pathologist decided to perform an internal autopsy (and to leave the corpse intact where he or she did not). In total, each informed consent procedure took approximately 20–30 min.

If the family spoke a language that could not be spoken by the researcher, a Forensic Pathology Officer would translate; as such, we were able to communicate all major languages spoken in the Western Cape. In one instance, a family spoke a dialect which none of the Forensic Pathology Officers could speak, and for fear of a misunderstanding, it was decided to not approach them for participation in the study. Similarly, if the consent process became too distressing for the family member, it was stopped immediately. In circumstances when religious leaders, such as the Imam, Rabbi or Hindu priest, accompanied the family to the mortuary, it was decided to include these religious leaders in the consent process to (i) show respect the family wishes and (ii) to ensure that members of these religious groups could also be included in the study if they wished.

The researcher in the first study is a forensic pathologist with over 10 years of experience and skill with dealing with bereaved families of people on a daily basis. During her specialist training, she developed the prerequisite skills to conduct consent for this research project. The researcher in the second study is a biomedical scientist who was specifically trained to talk to families in stressful and emotional situations. Training took the form of observations, role plays and debriefing sessions conducted by a team of trained medical professionals, until the researcher was ready to take consent on her own.

At the mortuary, managers can provide relief grief counselling and support. If this was not adequate then religious leaders were contacted to provide support. One suggestion that emerged from our experiences was to implement a better referral system for family members to speak to qualified grief counsellors in their area, or at Groote Schuur hospital, for a longer time after the identification process, which could be arranged on a case by case basis.

### Vulnerability: Recently bereaved individuals under study

South African laws and ethical guidelines define several kinds of vulnerable groups that require special consideration in research and beyond. In relation to research, the Department of Health Ethical Guidelines (2015) define vulnerable individuals as those whose willingness to participate in research might be influenced either by an expectation of benefit or by peer pressure [18]. In the South African legislative and regulatory framework, identified vulnerable groups include children, mentally ill or disabled individuals, prisoners, subordinate members of hierarchies, poor people, patients in emergency situations and ethnic and racial minorities [19]. Neither the South African ethical nor the legal frameworks that describe or identify vulnerable groups explicitly list deceased individuals and their living relatives as vulnerable.

The Belmont Report (1979) introduced the idea of “diminished autonomy”, whereby vulnerable groups could be identified as those with a “dependent status” and “inability or limited capacity to provide free consent” [20]. In the case of deceased individuals, whether they are children or adults, their capacity to provide consent is non-existent, and they are completely ‘dependent’ on family members or their next of kin to provide informed consent. But the important question is whether the next of kin should not also be considered a vulnerable group. In a period of profound grief, the next of kin is given the added responsibility of making a decision with regards to consent for samples to be taken from their child/family member/friend to be used for research. Such individuals may or may not have a “dependent status”; for example they might be subordinate members of hierarchies, they might be poor individuals or they might be from ethnic and racial minorities. In these instances, they are vulnerable in one sense, but does the fact that they are grieving make this population a vulnerable group in their own right? While grief does not strictly match the definition of a vulnerable group, in our experience the capacity of grieving individuals to freely give consent may be limited due to various reasons including:

(i) The experience of shock and grief, both of which could have impaired consenting adults’ ability to understand the information provided;
(ii) An expectation that they ought to do everything possible for their deceased and therefore give consent without fully considering all aspects of the research;
(iii) Pressures from their community to do what is deemed ‘best’; this could be influenced by religion, culture and what is perceived to be society’s norm;
(iv) They themselves want to know what happened (peace of mind) and a molecular diagnosis could relieve them from feeling guilty and/or defer them blaming themselves for the death;
(v) Fear of what the results might reveal about their parenting, in the case of SUDI cases or interpersonal issues in the case of suicide.

From our perspective, the research participant is the deceased individual as the biological sample was taken from them; however, consent was given by their next of kin, who – we propose – should be considered as vulnerable in their own right due to their state of grief. Therefore, the current definition of vulnerability is perhaps too narrow, as it does not cater for other vulnerable groups who are not participants themselves, but who are approached for consent during a time in which they are vulnerable in the same way that participants would be.

There is considerable discussion in bioethics on how vulnerability should be understood and accounted for in research ethics [21]. What seems clear, however, is that if vulnerability is understood as potentially impairing an individual’s ability to give voluntary and informed consent, then there is an argument for ‘special arrangements’ to be put in place that promote the vulnerable person’s ability to understand the information, and give consent without undue pressure. This could, for instance, involve deferral or progressive staging of consent, where individuals are approached for initial consent but then are approached for re-consenting when the factors causing their vulnerability have resolved. A pertinent example for our discussion is the enrolment of paediatric emergency patients in research [22, 23]. The categorisation as a particular group of participants as ‘vulnerable’ thus seems to open up the possibility that consent processes more explicitly take into account the circumstances of the person consenting. In our experience, consenting grieving adults for enrolment in research, when they have just lost a family member or child and are awaiting autopsy is a really challenging, if not impossible, task and that we should carefully consider more appropriate ways of seeking consent from surviving relatives, possibly over time.

A key feature of discussions around vulnerability is that the exclusion of vulnerable groups from research merely because it is inconvenient may be considered unethical [18, 19]. Instead, research should also target vulnerable groups, but greater care should be taken to specify the best interests of the population, and only research that offers a direct benefit to the population against acceptable risk should be conducted. It emerges that a clear benefit for both the deceased population as well as the family members needs to be clearly defined in the research study.

Regarding the consenting family members as a vulnerable group also highlights the need to ensure that they are satisfied with the consent and research study over time. Specifically, because of concerns about the consenting individual’s state of mind at the time of consent, we think it is imperative to engage with those same family members after some time has elapsed, to ensure they are still satisfied with the consent they gave, have any questions about the samples, and to give an update on research progress.

While working with these vulnerable groups has generated concerns about potential exploitation, we have also experienced a reaction of ‘empowerment’ by the next of kin. Often, family members who are left behind seem to blame themselves for the death, wonder what they could have done to prevent the death and feel responsible for what happened. They feel very helpless following a death. The opportunity to participate in our projects has, at times, given family members a sense of hope and empowerment in that their family member may not have died in vain, but could be seen as helping others, through this research. The feeling of empowerment has been reported before in other studies including vulnerable populations [24]. Hence, it is important to appreciate the full spectrum of reactions from family members and consequently to design mortuary-based projects with these reactions/feelings/emotions in mind.

### Researcher’s emotional skills: Pressures of approaching family members for conducting research

Research on this population is challenging and it places considerable pressure on researchers, as speaking to grieving family members about a research study is emotionally and practically challenging for various reasons. These include feelings of instability and uncertainty in order to be available at unpredictable times to go to the mortuary and meet the family, preparation for a wide spectrum of responses from the family (individuals may exhibit a blunted affect, be angry, crying or hysterical), possessing skills to provide articulate information about the research, as well as interview skills, whilst exhibiting strength, patience, sympathy, adaptability, commitment, knowledge, insight and credibility. Speaking to family members who have just lost an infant, for instance, is exceptionally taxing and debriefing after every session is essential for researchers who find themselves in similar situations.

Because of the challenges relating to seeking consent from recently bereaved family members, we consider the following elements of good practice:

(i) Preference should be for individuals with prior training in the forensic autopsy setting to seek informed consent for such research;
(ii) Thorough training in how to communicate about the research study is essential before researchers are left to their own devices. Such training is labour- and time-intensive and needs to be done carefully and sensitively. For this reason, it is unlikely that students or short-term research staff can be trained appropriately and these should only be involved if they already have some of the required skills or experience necessary to communicate with grieving family members appropriately;
(iii) Counselling support for researchers is essential to help them develop coping mechanisms for dealing with the effects of repeatedly engaging with the trauma of families who have recently lost a family member or infant;

Ideally, there should be a designated person at the mortuary for all research studies but this may not be feasible.

### Study feedback: Sharing results with family members under study

When designing these studies, one important ethical question that we considered was whether we should share individual genetic research results with family members; and if so, what results would be fed back. It was decided that during the research phase of the studies, information will not be fed back to the families. This was decided upon for various reasons; to work out project logistics, burdens and hindrances; to prevent families from revisiting the trauma in what may be the distant future; as well as due to the lack of clarity regarding what pathogenic mutations existed in the local populations [25]; in other words the initial pilot research was intended to be exploratory. Once clinically significant mutations have been shown to underlie suicide and SUDI, it is intended that a screening molecular assay will be set up and validated to be offered prospectively as part of the post-mortem investigation. In this instance, it is envisaged that a genetic counsellor would be involved to explain the significance of the genetic results with the family. However, it is also possible that aspects of genetic counselling may be introduced into the repertoire of training of forensic pathologists and scientists involved with molecular autopsies.

It is, however, imperative to make it clear in the informed consent process, that the family members will not receive feedback by participating in this research study. It should also be explained that the study is unlikely to benefit them or their family directly, but it is hoped that it will contribute to knowledge about suicide or SUDI in the future.

However, an unresolved challenge in our study is that of deciding what to do if, during exome sequencing of the deceased individual’s DNA, an *unrelated* mutation is detected which would have implications on the living family. An example would be a *BRCA2* mutation which is likely to have been inherited from the parent. The ethical question is whether to inform the family of this mutation or not? We have tentatively established a policy that would allow us to return such findings if and only if three criteria are met: a) the mutation must be medically actionable; b) it must be unlikely that the condition would have been diagnosed without the genetic finding, and c) the mutations must have been validated across multiple studies that are relevant to the South African population [25]. Although we have not yet come across this, it is important to consider what would be done in such a case in the future. The anticipated challenges here are manifold, but include: the funding of validation studies, the availability and funding of a genetic counsellor to feedback information to the family, and also the challenge of contacting the bereaved family, again, and with further ‘bad news’. These conditions and limitations must be communicated to the family during the initial, already challenging consent process, which at this stage is exploring a wide range of issues. It is likely that, as the project progresses, and the research team develops, that the issue of unexpected findings may be dealt with more comprehensively.

On another level, because of the vulnerability of the consenting participants and because family members – and particularly parents of SUDI – may never forget about their child’s sample sitting in a laboratory somewhere, it is imperative that researchers are conscious of the need for ongoing communication about study progress and generalised study findings. This could take the form of e.g. an annual meeting for all research participants (which may have the added benefit of bringing together people with similar experiences), individual genetic counselling sessions, or phone calls if and when the project reveals new and important findings. In our study, we intend to use a mix of all three approaches.

## Conclusions

In order to accommodate these ethical concerns, we have developed a number of practices that aim to ensure forensic genetic studies are conducted without causing unnecessary upset. These include some practices that are part of good practice for research generally, and others which are specific to conducting research on deceased individuals. With regard to the first, it is imperative that researchers are transparent about the study – no matter how difficult it is to speak with recently bereaved individuals. It is also essential to provide contact numbers for the researchers, the ethics committee and also for any support services that are available. Considering the potential vulnerability of the family members, maintaining confidentiality is key, as is providing information about the study in the next of kin’s own language. Recruitment processes need to be flexible enough to allow a multitude of individuals to be involved in deciding on research participation. This includes not only multiple family members, but also, for instance, religious leaders who accompany the family to the mortuary.

Specific to research being conducted on deceased individuals, we learned that it is important to provide study information within the identification process, before family members view the infant or suicide victim. It is also important to ensure that the consent process takes place in a suitable environment that is quiet and private – and many mortuaries would have such suitable spaces. Considering the emotional burden of working in these environments, it is imperative that researchers not only receive appropriate training to deal with the range of emotions they may encounter, but that they also receive counselling support to deal with any issue or stresses arising from this process. In addition, we advocate that projects should consider budgeting for grief counselling support for family members, particularly considering that such services are overstretched in South Africa and likely to be so in other LMICs. Projects should also budget for ongoing information meetings with participants enrolled in the research projects, to update family members on project progress and to reiterate the information provided during the consent process. We would also advocate that ‘forensic genetic counselling’ is added to the training curriculum of genetic counsellors and potentially other medical genetics professionals.

We hope that our experiences will contribute to the development of a framework for conducting research in mortuaries in South Africa, especially as we get a better understanding of the contribution of genetic factors to death. This paper will feed into a meeting that we are hoping to organise with relevant stakeholders from across the country. During that meeting, we will aim to discuss the ethical challenges of doing mortuary research, gain broad input on the ways in which these challenges could be addressed, and identify points of consensus that can form the basis of a common approach to mortuary research.

## Abbreviations

DFMT: Division of Forensic Medicine and Toxicology
DNA: Deoxyribonucleic acid
FPS: Forensic Pathology Service
LMIC: Low- and middle- income countries
SIDS: Sudden infant death syndrome
SUDI: Sudden unexpected death in infants
UCT: University of Cape Town
